# A fast method to distinguish between fermentative and respiratory metabolisms in single yeast cells

**DOI:** 10.1016/j.isci.2023.108767

**Published:** 2023-12-21

**Authors:** Laura Luzia, Julius Battjes, Emile Zwering, Derek Jansen, Chrats Melkonian, Bas Teusink

**Affiliations:** 1 Systems Biology Lab, A-LIFE, Institute of Molecular and Life Sciences (AIMMS), VU Amsterdam, 1081HZ Amsterdam, the Netherlands; 2 Theoretical Biology and Bioinformatics, Department of Biology, Faculty of Science, Utrecht University, Utrecht, the Netherlands; 3 Bioinformatics Group, Wageningen University and Research, 6700AP Wageningen, the Netherlands

**Keywords:** Microbial biotechnology, Cell biology

## Abstract

*Saccharomyces cerevisiae* adjusts its metabolism based on nutrient availability, typically transitioning from glucose fermentation to ethanol respiration as glucose becomes limiting. However, our understanding of the regulation of metabolism is largely based on population averages, whereas nutrient transitions may cause heterogeneous responses. Here we introduce iCRAFT, a method that couples the ATP Förster resonance energy transfer (FRET)-based biosensor yAT1.03 with Antimycin A to differentiate fermentative and respiratory metabolisms in individual yeast cells. Upon Antimycin A addition, respiratory cells experienced a sharp decrease of the normalized FRET ratio, while respiro-fermentative cells showed no response. Next, we tracked changes in metabolism during the diauxic shift of a glucose pre-grown culture. Following glucose exhaustion, the entire cell population experienced a progressive rise in cytosolic ATP produced via respiration, suggesting a gradual increase in respiratory capacity. Overall, iCRAFT is a robust tool to distinguish fermentation from respiration, offering a new single-cell opportunity to study yeast metabolism.

## Highlights

• iCRAFT is a single-cell method to distinguish fermentative from respiratory metabolisms
• During the diauxic shift cells simultaneously switch from glucose to ethanol consumption
• Yeast respiratory capacity uniformly increases after the diauxic shift

## Graphical abstract

**Figure:**
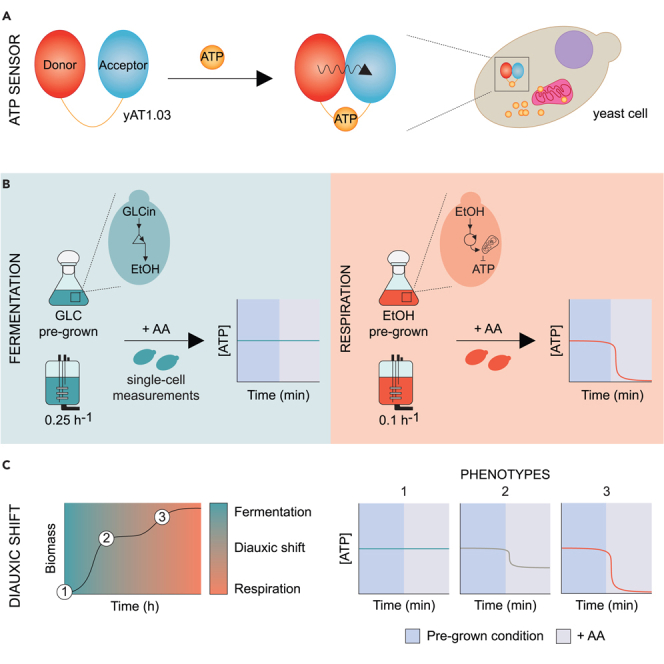

## Introduction

Adenosine triphosphate (ATP), universally regarded as the main energy currency of life, is involved in essential cell processes such as growth, intracellular transport, and regulation of central carbon metabolism.^1^^,^^2^^,^^3^^,^^4^^,^^5^ Cells rely on catabolic processes to synthesize ATP, either through respiration and proton motive force-driven ATPase or through fermentation and substrate-level phosphorylation. *Saccharomyces cerevisiae* (*S. cerevisiae*) and other Crabtree-positive yeast ferment glucose to ethanol in the presence of oxygen.^6^^,^^7^^,^^8^ When glucose is exhausted, cells switch to ethanol respiration, generating additional ATP. This transition is known as the diauxic shift (DS) and involves extensive remodeling of the cell metabolome and proteome.^9^^,^^10^ Among those are the expansion and extensive reshaping of mitochondria that create an intricate tubular network enabling fully respiratory growth.^11^^,^^12^

Proteomic changes in yeast cell makeup toward respiratory metabolism were shown to start gradually in the mitochondria, when glucose is still available.^10^ It is still unclear if this gradual shift is caused by a gradual shift in each individual cell, or a population average artifact of heterogeneous but sudden shifts at the single-cell level. A few studies have investigated single-cell responses of metabolism. van Heerden used intracellular pH to follow metabolism during startup of glycolysis;^13^ similarly, Bagamery used mitochondrial abundance and morphology to assess metabolic states after glucose starvation and recovery^14^; Takaine measured ATP in mutants of ATP metabolism and linked it to protein aggregation.^15^ None directly assessed the functional contribution of respiration versus fermentation to ATP homeostasis and the cell heterogeneity during the DS. Techniques that come closest to assess mitochondrial function are based on measuring changes in mitochondrial membrane potential and include the use of fluorescent dyes or an oxygraph together with uncoupling agents in intact cells or isolated mitochondria.^16^^,^^17^ While the former method is known for its inability to respond to fast changes in membrane potential,^18^ the latter lacks single cell resolution (see Table S1 for comparison of methods).

Here, we established a novel single-cell method to directly and quickly distinguish between respiration and fermentation as the dominant mode of ATP production in single yeast cells-iCRAFT: individual cell respiration and fermentation tracker. iCRAFT relies on the expression of the Förster resonance energy transfer (FRET) bio-based sensor yAT1.03^19^ to measure cytosolic ATP changes upon addition of the respiration blocker Antimycin A (AA). After being pulsed with AA, respiratory cells undergo a reduction in ATP levels as a result of the impairment of the electron transport chain,^20^ whereas cells that ferment maintain the original values. We then applied iCRAFT to a yeast population during the DS to test for the presence of potential metabolic subpopulations.

## Results

### Induction of ATP drain in fermentative and respiratory cells after a pulse with a glucose analogue

In this work we used the bio-based FRET sensor yAT1.03 to measure changes in cytosolic ATP in CEN.PK. In parallel, the strain CEN.PK + ymTq2Δ11 was generated (Table 1 and Figure S1A) to correct for bleedthrough of the acceptor (ymTq2Δ11) into the donor channel (tdTomato) (Figures S1B and S1C), accounting for possible differences in fluorescence emission when compared with CEN.PK + ymTq2. When expressed in CEN.PK, yAT1.03 does not compromise growth; however, a slight decrease in growth rate under ethanol conditions and final biomass was observed (Figure S2).

**Table 1:** Yeast strains and plasmids

Strain background	Plasmid	Fluorescent protein(s)	Strain short name	Reference	Source
CEN.PK113-7D	–	–	7D	–	–
CEN.PK113-5D	pDRF1-GW	–	5D	W. Frommer	Addgene #36026
CEN.PK113-5D	yAT1.03 pDRF1-GW	ymTq2Δ11, tdTomato	yAT1.03	D. Botman	Addgene #132781
CEN.PK113-5D	yAT1.03R122KR126K pDRF1-GW	ymTq2Δ11, tdTomato	yAT1.03NR	D. Botman	Addgene #132782
CEN.PK113-5D	tdTomato pDRF1-GW	tdTomato	tdTomato	D. Botman	In-house
CEN.PK113-5D	ymTq2 pDRF1-GW	ymTq2	ymTq2	D. Botman	Addgene #118453
CEN.PK113-5D	ymTq2Δ11 pDRF1-GW	ymTq2Δ11	ymTq2Δ11	L. Luzia	Addgene #205490

**Figure 1:**
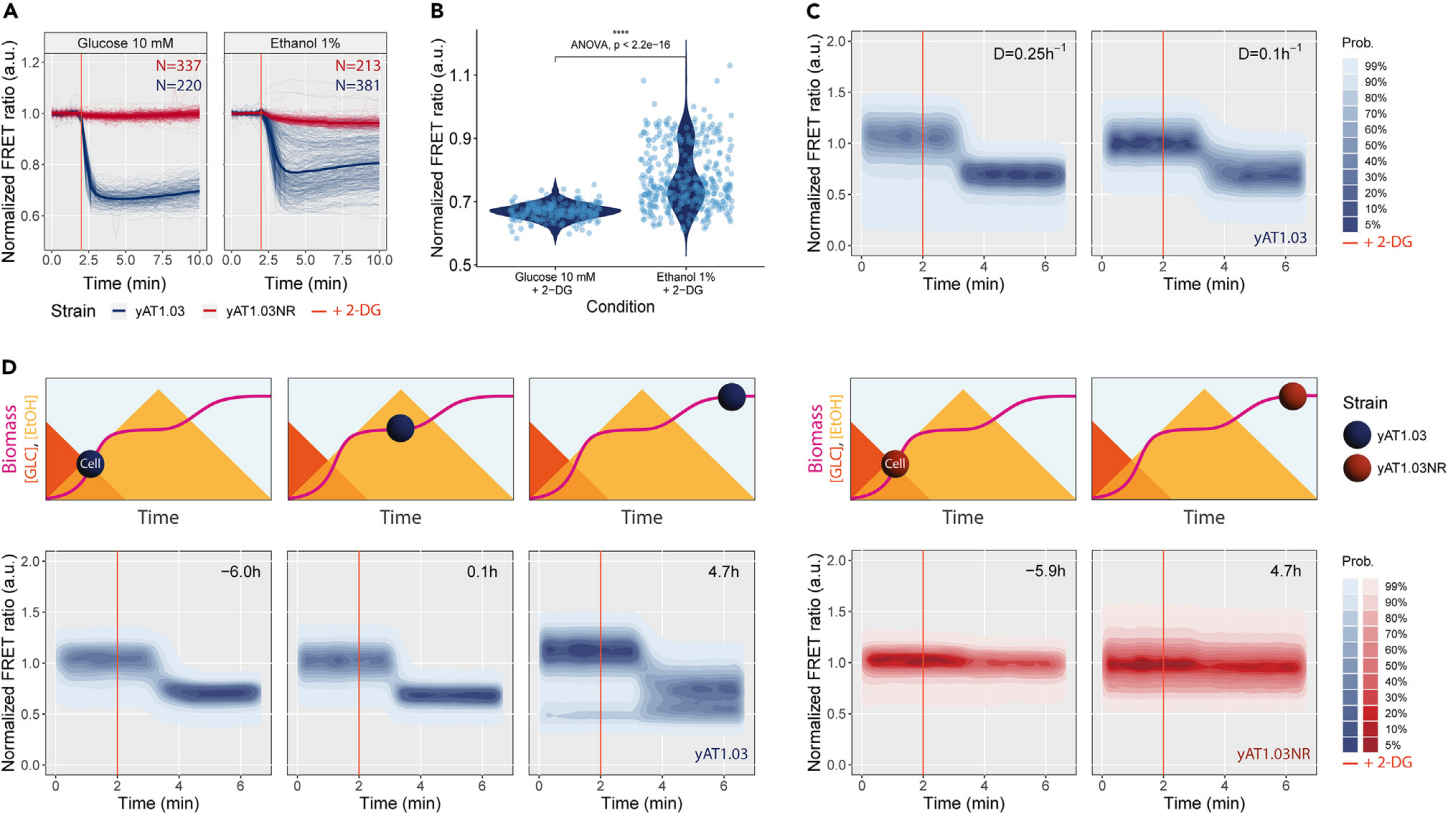
Decrease in intracellular ATP triggered by an ATP drainer during fermentatition, respiration, and the diauxic shift (A and B) Cells expressing the responsive (yAT1.03) or the non-responsive (yAT1.03NR) sensors were incubated in minimal media supplemented with 10 mM glucose or 1% ethanol followed by a pulse with 50 mM of 2-Deoxy-D-glucose (2-DG). The ATP dynamics were measured by fluorescent microscopy. (A) Thin lines illustrate single-cell trajectories (N number of cells) and thick lines the mean FRET ratio normalized to the baseline. (B) Violin plots of the means of yAT1.03 displayed in (A) measured between 3.5 and 4.5 min. The statistical tests Mann-Whitney U (p value <0.0001) and analysis of variance (ANOVA) were applied to the data. (C) ATP changes measured by flow cytometry upon 2-DG addition to yAT1.03 cells pre-grown in an aerobic controlled stepwise chemostat (D = 0.1 and 0.25 *h*^−1^). (D) ATP dynamics measured by flow cytometry after 2-DG addition to cells expressing yAT1.03 (pre-, during, and post-diauxic shift) and yAT1.03NR (pre- and post-diauxic shift). The sampling time is indicated in the upper right corner of each subplot. Top diagrams illustrate the metabolic state of the cell, from growth on glucose (GLC), through the diauxic shift, to growth on ethanol (EtOH). Spheres represent yeast cells, and their colour code the strain: blue yAT1.03 and red yAT1.03NR.

We started by testing the suitability of the ATP sensor yAT1.03 by pulsing cells with an ATP drainer that should result in a decrease in cytosolic ATP in both fermentative and respiratory regimes. To do so, we submitted a population of yeast cells pre-grown on either ethanol or glucose to the glycolysis inhibitor 2-Deoxy-D-glucose (2-DG) (concentration selected based on a dose-response curve can be found in Figure S3). Cells grown on both carbon sources experienced a drop in ATP concentration upon 2-DG addition, and no recovery was observed 8 min after the pulse (Figure 1A). Quantitatively we observed a more heterogeneous response for cells grown on ethanol (Figure 1B).

Next, we applied the same perturbation to a culture grown in a chemostat at 0.1 and 0.25 $h^{-1}$ dilution rates, aiming to achieve respiratory and respiro-fermentative metabolisms when providing the same carbon source.^21^ The chemostat data show that the cultures transitioned indeed from a respiratory to a fermentative state (ethanol production rates of 0 $mmol\;g_{DW}^{-1}$ $h^{-1}$ at 0.09 $h^{-1}$ and 7 $mmol\;g_{DW}^{-1}$ $h^{-1}$ at 0.26 $h^{-1}$, respectively) (Table 2). 2-DG addition resulted again in a drop in cytosolic ATP levels for both dilution rates (Figure 1C, full data in Figure S4). In parallel, we monitored the glucose concentration of the samples used in the pulse experiment. Despite very low glucose concentrations, as expected, ATP levels remained responsive to 2-DG addition (Figure S5; Table S2).

**Table 2:** Chemostat cultivations show dilution rate-dependent respiratory and fermentative metabolisms

Variable	D = 0.1 *h*^*−*^*^1^*	D = 0.25 *h*^*−*^*^1^*
*Growth rate (h*^*−*^*^1^)*	0.09 ± 0	0.26 ± 0
*Y*_*x/s*_*(g*_*DW*_*.g*^*−*^*^1^*_*glucose*_*)*	0.42 ± 0	0.20 ± 0.03
*q*_*ethanol*_*(mmol.g*^*−*^*^1^*_*DW*_*.h*^*−*^*^1^)*	0	6.95 ± 1.34
*q*_*glucose*_*(mmol.g*^*−*^*^1^*_*DW*_*.h*^*−*^*^1^)*	−1.25 ± 0.01	−7.06 ± 0
*q*_*glycerol*_*(mmol.g*^*−*^*^1^*_*DW*_*.h*^*−*^*^1^)*	0	0
*q*_*pyruvate*_*(mmol.g*^*−*^*^1^*_*DW*_*.h*^*−*^*^1^)*	0	0
*q*_*succinate*_*(mmol.g*^*−*^*^1^*_*DW*_*.h*^*−*^*^1^)*	0	0
c_*glucose,out*_*(mmol.l*^*−*^*^1^)*	0.01 ± 0	0.09 ± 0.01
c_*ethano*__*l*__*,out*_*(mmol.l*^*−*^*^1^)*	0	10.17 ± 0.63

Cells grown in glucose media at a dilution rate of 0.1 $h^{-1}$ display a respiratory behavior while cells grown at a dilution rate of 0.25 $h^{-1}$ display a fermentative behavior. Data originated from biological duplicates. Yxs is yield of biomass on glucose; *q* is specific flux with negative values meaning consumption; *c* is concentration measured in the final broth.

Finally, we followed the response to 2-DG addition during the DS. Pre- and post-DS cells equally experienced a drop in ATP concentration after the perturbation, but again growth on ethanol appeared to give a more heterogeneous response (Figure 1D). No response was detected for the non-responsive sensor (yAT1.03NR), here used as a control.

### Disruption of mitochondrial ATP synthesis allows to distinguish between fermentative and respiratory metabolisms

**Figure 2:**
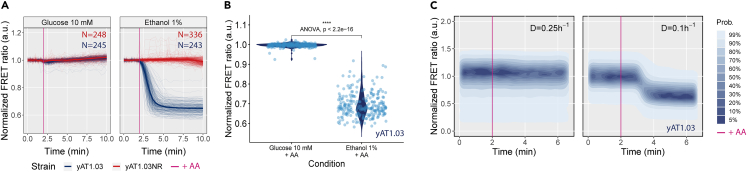
ATP dynamics upon Antimycin A addition in respiratory and fermentative cells reveal alternative metabolims (A and B) Cells expressing yAT1.03 or yAT1.03NR incubated in minimal media supplemented with 10 mM glucose or 1% ethanol were pulsed with 50 μM of Antimycin A (AA), and fluorescence was followed by microscopy. (A) Thin lines illustrate the single-cell trajectories (N number of cells) and thick lines the mean FRET ratio normalized to the baseline. (B) Violin plots of the means of yAT1.03 displayed in (A) measured between 3.5 and 4.5 min. The statistical tests Mann-Whitney U (p value <0.0001) and ANOVA were applied to the data. The same experiment was repeated using flow cytometry, in two independent experiments. (C) ATP changes upon AA addition to yAT1.03 cells pre-grown in an aerobic controlled stepwise chemostat (D = 0.1 and 0.25 *h*^−1^) measured by flow cytometry.

Earlier computational work had shown that yeast cells grown on glucose excess rely mostly on substrate-level phosphorylation for ATP synthesis: only 20% of total ATP production flux comes from respiration.^21^ We therefore wondered if we could distinguish fully respiratory growth from respiro-fermentative growth by inhibiting respiration and monitoring the response to cytosolic ATP levels. We then applied iCRAFT to a yeast cell population expressing yAT1.03 by submitting it to AA, an inhibitor of complex III of the respiratory chain. Using both microscopy and flow cytometry, we observed a decrease in ATP levels in ethanol pre-grown cells upon AA addition, but no change in glucose pre-grown cells (microscopy data displayed in Figures 2A and 2B and flow cytometry data in Figure S6).

Next, we turned to aerobic glucose-limited chemostat conditions, where *S. cerevisiae* remains respiratory at low dilution rates and becomes fermentative at high dilution rates. Cells growing at 0.1 $h^{-1}$ suffered a drop in ATP levels after pulsed with AA, whereas no effect was detected for cells growing at 0.25 $h^{-1}$ (Figure 2C; full data in Figure S7). No subpopulations were observed in any of the experiments.

### ATP dynamics during the DS reveal distinct respiratory capacities

**Figure 3:**
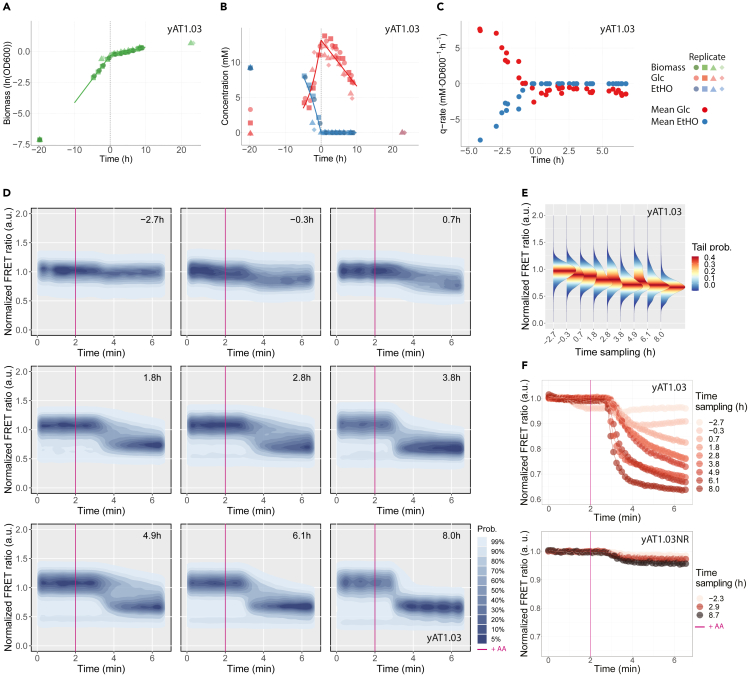
ATP dynamics during the diauxic shift reveal distinct respiratory capacities Cells expressing yAT1.03 or yAT1.03NR were grown in minimal media supplemented with 10 mM glucose. (A) Growth profile. (B) Glucose and ethanol concentrations. (C) Glucose and ethanol biomass specific rates (replicates pooled together). (D) Normalized ATP FRET ratio response to a 50 μM pulse of Antimycin A (AA) measured by flow cytometry. Data normalized to the baseline. The sampling time is indicated in the upright corner of each subplot. (E) Distribution plots of (D) after the effect of AA becomes visible (3.33 min from the start). (F) Binned plots of the ATP FRET ratio (mean data of 50 consecutive time intervals) for yAT1.03 and yAT1.03NR sensors. Plots (A), (B), and (C) include data of four biological replicates and plots (D), (E), and (F) data of a representative experiment.

Finally we followed the changes in fermentation versus respiration in individual cells during the DS. We sampled a glucose batch culture expressing yAT1.03 over time and pulsed cells with AA. Additional samples were collected to measure growth and metabolites concentration (Figures 3A and 3B). The population-based specific rates of glucose uptake and ethanol excretion/uptake were then calculated (Figure 3C). Altogether the data allowed us to define the time window corresponding to the DS and thus the pre- and post-DS phases. The pre-DS phase spanned for 20 h, from inoculation until glucose depletion. This phase was followed by the post-DS phase characterized by ethanol consumption and a decrease in growth rate from 0.37±0.05 to 0.08±0.00 $h^{-1}$. After being pulsed with AA, pre-DS cells showed no change in ATP FRET ratio, whereas post-DS cells experienced a decrease in the ratio (Figure 3D). During the 8 h that followed the DS, we observed an increasing drop in ATP levels until it reached 0.6 (density values after the effect of AA become visible in Figure 3E and mean values in Figure 3F; replicates data in Figure S8). At this point, cells are fully respiratory and have consumed 55% of the original ethanol available. No physiological subpopulations were spotted throughout the experiment, with exception of a fraction of cells with a low FRET ratio that becomes visible 2.8 h after the DS and that dominates after ethanol exhaustion (Figure S9), suggesting the presence of dead cell material. No response was observed for the non-responsive sensor (yAT1.03NR) after AA addition in the different stages of growth. The expression levels of the donor and acceptor proteins and their relationship with the non-normalized FRET ratio can be found in Figure S10.

## Discussion

In this work we developed a direct method to distinguish fermentative from respiratory metabolism in *in vivo* single cells of *S. cerevisiae*. The novel method is based on the expression of the fluorescent ATP sensor yAT1.03 and short-term perturbations with the respiration inhibitor Antimycin A. We validated the method in fully fermentative and respiratory metabolic regimes from batch and chemostat cultures. Finally, we used iCRAFT to follow the diauxic transition from glucose fermentation to ethanol respiration in a yeast cell population.

The glucose analogue 2-Deoxy-D-glucose was used in a control experiment to induce expected changes in cytosolic ATP levels regardless of the carbon source provided (Figure 1). 2-DG is imported into the cytosol by hexose transporters and phosphorylated by hexokinase into 2-Deoxy-D-glucose-6-phosphate (2-DG-6P), in a step that requires one ATP molecule. The lack of the hydroxyl group in the backbone of 2-DG-6P makes it unrecognizable by glucose-6-phosphate isomerase, resulting in a blockage of glycolysis.^22^ In glucose-grown cells, 2-DG competes with glucose, directly disrupting ATP synthesis and rapidly depleting ATP. In ethanol-grown cells, metabolism is fully geared toward gluconeogenesis and respiration, not glycolysis. One can therefore expect that the capacity to take up and phosphorylate 2-DG is relatively low and apparently different between individual cells. Considering that glycolysis is downregulated during respiration,^10^^,^^23^^,^^24^ the observed ATP drop in this regime confirms the preparedness of yeast to catabolize its preferred carbon source, glucose. Indeed, metabolic anticipation has been previously demonstrated in budding yeast.^25^^,^^26^ The great heterogeneity observed in the response of ethanol-grown cells to 2-DG might arise from variable expression of proteins involved in glucose sensing and import, glycolysis startup, or signaling pathways related to glucose repression such as Mig1-Hxk2. While this remains a hypothesis, we lack single-cell evidence to pinpoint the specific agents involved. The fitness benefit of cell-to-cell heterogeneity in response to unpredictably changing environments, also known as bet-hedging, is well known in yeast^14^^,^^27^^,^^28^^,^^29^ and is expected to play a higher effect at lower growth rates.^30^^,^^31^

AA acts by impairing the mitochondrial electron transport chain complex III (coenzyme Q: cytochrome *c* – oxidoreductase),^20^ compromising ATP synthesis through oxidative phosphorylation. The effect of a sudden block of respiration on the ATP concentration depends on the relative contribution of respiration to the overall ATP synthesis flux, and the flexibility of either substrate-level phosphorylation to take over ATP demand or ATP demand to lower in view of the limited supply. We tested both ethanol-grown cells (no option at all to scale up substrate-level phosphorylation) and cells grown fully respiratory on glucose (at a low dilution rate, little substrate is available to respond to a drop in ATP). In both cases, with more than 90% of ATP coming from respiration and no backup from glycolysis, AA caused a drop in ATP. As expected, the ATP levels of fermenting cells remained constant after AA addition, both for glucose batch and chemostat at 0.25 $h^{-1}$ cultures (Figure 2A and 2B), despite low glucose levels in the latter case. Yeast cells growing on excess glucose rely on glycolysis for 75% of their ATP generation, and therefore a temporary drop in ATP upon inhibition of respiration could have been expected but was not observed even though we estimate that 25% of ATP supply runs via respiration.^32^ This may be explained by a rapid and sensitive activation of glycolysis, likely via AMP-mediated activation of phosphofructokinase. We expect that the inhibition by AA and the response of glycolysis through such metabolic regulation can act at the same time scales and render ATP homeostatically regulated. Moreover, glycolysis is responsive to ATP demand^13^ and under glucose excess has the ability to accelerate, which may be considered the reverse of the Pasteur effect.^33^

For the chemostat sample at a D = 0.25 $h^{-1}$, respiration cannot have contributed much to ATP homeostasis as a fast response of glycolysis is ruled out by the low glucose levels under these conditions. Yet, the sharp differences observed in the ATP pattern for respiratory and fermentative cells provide a clear separation of the two metabolic phenotypes required for the method design.

After establishing the method, we applied it to a yeast cell population transitioning from glucose fermentation to ethanol respiration during the diauxic growth. We found that, during the lag phase following glucose exhaustion, yeast cells progressively increased their respiratory capacity (gradual increase in ATP drop). Previous works linked the raise in respiratory capacity with the expansion of the number of mitochondria and its functionality.^14^ During the time course of the experiment no subpopulations were identified, indicating that all cells coordinate their response toward the DS, and the change in the slope and possibly end state of ATP (Figures 3F and S11) reflect changes in average capacities. One may expect that gradual and predictable changes in the environment should result in uniform responses, but there are notable exceptions, at least for prokaryotes.^34^

Altogether, our results show that iCRAFT is a robust method to distinguish fermentative from respiratory metabolisms in budding yeast. The method allows *in vivo* and single-cell measurements, providing a versatile platform that can be used in multiple growth setups (batch, chemostat, microfluidics) and instruments (plate reader, flow cytometer, microscope). Moreover, the short duration of the assay makes it a powerful method for quick measurements. iCRAFT brings new possibilities to study yeast metabolism under more realistic conditions often found in the biotechnology industry, such as in poorly stirring bioreactors and fermented products.

### Limitations of the study

In this study, we focused on fully fermentative and fully respiratory carbon sources. Exploring respiro-fermentative carbon sources, such as galactose, would be interesting for future research. Focusing now on the limitations of the method itself, while highly effective, iCRAFT comes with a few considerations. Firstly, the method involves the use of a mitochondria uncoupler, making it a destructive method and potentially affecting downstream analyses. Secondly, the method has only been tested in *S. cerevisiae*, remaining unclear about its general applicability in other species. Last, the method requires the expression of a DNA plasmid, which could be potentially circumvented by genomic integration.

## STAR★Methods

### Key resources table

**Table:** 

REAGENT or RESOURCE	SOURCE	IDENTIFIER
**Bacterial and virus strains**		

*E. coli* DH5-alpha competent cells (High Efficiency) + SOC media	New England Biolabs, Ipswich, Massachusetts, USA	Cat# C2987I

**Chemicals, peptides, and recombinant proteins**		

2-Deoxy-D-glucose	Sigma-Aldrich, St. Louis, MO, USA	CAS: 154-17-6
Agarose SeaKem LE agarose	Lonza Netherlands B.V.	Cat# 50004
Ammonium sulfate	Carl Roth, Karlsruhe, Germany	CAS: 7783-20-2
Ampicillin	Duchefa, Haarlem, The Netherlands	CAS: 69-52-3
Antifoam C	Sigma-Aldrich, St. Louis, MO, USA	Cat# 1003584422
Antimycin A from Streptomyces sp.	Sigma-Aldrich, St. Louis, MO, USA	CAS: 1397-94-0
Bacto peptone	Becton Dickinson, Franklin Lakes, NJ, USA	Cat# 211677
Biotin	Sigma-Aldrich, St. Louis, MO, USA	CAS: 58-85-5
Boric acid	Duchefa, Haarlem, The Netherlands	CAS: 10043-35-3
Calcium chloride dihydrate	J.T.Baker, Phillipsburg, NJ, USA	CAS: 10035-04-8
Calcium chloride dihydrate	J.T.Baker, Phillipsburg, NJ, USA	CAS: 10035-04-08
Calcium pantothenate	Sigma-Aldrich, St. Louis, MO, USA	Cat# 2316
Cobalt chloride hexahydrate	J.T.Baker, Phillipsburg, NJ, USA	CAS: 7791-13-1
Concanavalin A from Canavalia ensiformis (ConA) Type IV, lyophilized powder	Sigma-Aldrich, St. Louis, MO, USA	CAS: 11028-71-0
Copper(II) sulfate pentahydrate	Sigma-Aldrich, St. Louis, MO, USA	CAS: 7758-99-8
D(+)-glucose-1-hydraat, z.z.	Boom BV, Meppel, Netherlands	Article: 76020761.5000
DL-Phenylalanine	Sigma-Aldrich, St. Louis, MO, USA	CAS: 150-30-1
dNTP Mix (10 mM each)	Thermo Fisher Scientific, Scientific, Waltham, MA, USA	CAS: R0192
Ethanol absolute	VWR International, Radnor, PA, United States of America	CAS: 64-17-5
Ethylenediaminetetraacetic acid (EDTA)	Sigma-Aldrich, St. Louis, MO, USA	CAS: 6381-92-6
G418	Santa Cruz Biotechnology Inc., Dallas, TX, USA	Cat# sc-29065
Hexokinase/Glucose-6-phosphate dehydrogenase	Roche, Vienna, Austria	Cat# 39085926
Inositol	Sigma-Aldrich, St. Louis, MO, USA	CAS: 87-89-8
Iron(II) sulfate heptahydrate, Ferrous sulfate heptahydrate	AnalaR | BDH Chemicals	CAS: 7782-63-0
L-Adenine	Sigma-Aldrich, St. Louis, MO, USA	CAS: 73-24-5
L-Alanine	J.T.Baker, Phillipsburg, NJ, USA	CAS: 56-41-7
L-Arginine	Sigma-Aldrich, St. Louis, MO, USA	CAS: 74-79-3
L-Asparagine monohydrate	Carl Roth GmbH & Co	CAS: 5794-13-8
L-Aspartic acid	Sigma-Aldrich, St. Louis, MO, USA	CAS: 56-84-8
LB media	Sigma-Aldrich, St. Louis, MO, USA	Product: L3022
L-Cysteine	Sigma-Aldrich, St. Louis, MO, USA	CAS: 52-90-4
L-Glutamic acid	Sigma-Aldrich, St. Louis, MO, USA	CAS: 56-86-0
L-Glutamine	Sigma-Aldrich, St. Louis, MO, USA	CAS: 56-85-9
L-Glycine	AppliChem GmbH, DE	CAS: 56-40-6
L-histidine	Santa Cruz Biotechnology Inc., Dallas, TX, USA	CAS: 71-00-1
L-Isoleucine	Duchefa, Haarlem, The Netherlands	CAS: 73-32-5
Lithium acetate dihydrate	Thermo Fisher Scientific, Scientific, Waltham, MA, USA	CAS: 6108-17-4
L-Leucine	Sigma-Aldrich, St. Louis, MO, USA	CAS: 61-90-5
L-Lysine	Sigma-Aldrich, St. Louis, MO, USA	CAS: 56-87-1
L-Methionine	Sigma-Aldrich, St. Louis, MO, USA	CAS: 63-68-3
L-Proline	Sigma-Aldrich, St. Louis, MO, USA	CAS: 14-85-3
L-Serine	Sigma-Aldrich, St. Louis, MO, USA	CAS: 56-45-1
L-Threonine	Sigma-Aldrich, St. Louis, MO, USA	CAS: 72-19-5
L-Tryptophan	Sigma-Aldrich, St. Louis, MO, USA	CAS: 73-22-3
L-Tyrosine	Sigma-Aldrich, St. Louis, MO, USA	CAS: 60-18-4
L-Valine	Duchefa, Haarlem, The Netherlands	CAS: 72-18-4
Magnesium sulfate heptahydrate	Sigma-Aldrich, St. Louis, MO, USA	CAS: 10034-99-8
Manganese(II) chloride monohydrate	Riedel-de Haen, Hanover, Germany	CAS: 64333-01-3
Manganese(II) chloride monohydrate	Sigma-Aldrich, St. Louis, MO, USA	CAS: 64333-01-3
Molybdic acid sodium salt dihydrate	Sigma-Aldrich, St. Louis, MO, USA	CAS: 10102-40-6
Monopotassium phosphate	Sigma-Aldrich, St. Louis, MO, USA	CAS: 7778-77-0
myo-Inositol	Sigma-Aldrich, St. Louis, MO, USA	CAS: 87-89-8
NheI HF	New England Biolabs, Ipswich, Massachusetts, USA	CAS: R3131S
Nicotinic acid	Sigma-Aldrich, St. Louis, MO, USA	CAS: 59-67-6
NotI HF	New England Biolabs, Ipswich, Massachusetts, USA	CAS: R3189S
Oxoid Yeast Extract Powder	Thermo Fisher Scientific, Scientific, Waltham, MA, USA	Cat# LP0021B
*para*-Aminobenzoic acid	Duchefa, Haarlem, The Netherlands	CAS: 150-13-0
Phusion Hot Start II DNA Polymerase (2 U/μL)	Thermo Fisher Scientific, Scientific, Waltham, MA, USA	CAS: F549S
PIPES	Sigma-Aldrich, St. Louis, MO, USA	CAS: 5625-37-6
Poly(ethylene glycol) mol wt 3350	Sigma-Aldrich, St. Louis, MO, USA	CAS: 25322-68-3
Potassium chloride	Sigma-Aldrich, St. Louis, MO, USA	CAS: 7447-40-7
Potassium hydrogen phthalate	Sigma-Aldrich, St. Louis, MO, USA	CAS: 877-24-7
Potassium hydroxide (pellets)	Sigma-Aldrich, St. Louis, MO, USA	CAS: 1310-58-3
Potassium Iodide	Sigma-Aldrich, St. Louis, MO, USA	CAS: 7681-11-0
Pyridoxine hydrochloride	Sigma-Aldrich, St. Louis, MO, USA	CAS: 58-56-0
Sodium chloride	Sigma-Aldrich, St. Louis, MO, USA	CAS: 7647-14-5
Sodium phosphate dibasic	Sigma-Aldrich, St. Louis, MO, USA	CAS: 7558-79-4
T4 DNA Ligase	Thermo Fisher Scientific, Scientific, Waltham, MA, USA	CAS: 15224017
Thiamine hydrochloride	Sigma-Aldrich, St. Louis, MO, USA	CAS: 67-03-8
Tris-Hydrochloride	Thermo Fisher Scientific, Scientific, Waltham, MA, USA	CAS: 1185-53-1
UltraPure Salmon Sperm DNA Solution	Thermo Fisher Scientific, Scientific, Waltham, MA, USA	CAS: 15632011
Yeast Nitrogen Base Without Amino Acids (YNB)	Sigma-Aldrich, St. Louis, MO, USA	SKU: Y0626-1KG
Zinc Sulfate Heptahydrate	Sigma-Aldrich, St. Louis, MO, USA	CAS: 7446-20-0

**Critical commercial assays**		

GeneJET Gel Extraction Kit	Thermo Fisher Scientific, Scientific, Waltham, MA, USA	CAS: K0691
GeneJET PCR Purification Kit	Thermo Fisher Scientific, Scientific, Waltham, MA, USA	CAS: K0701
GeneJET Plasmid Miniprep Kit	Thermo Fisher Scientific, Scientific, Waltham, MA, USA	CAS: K0502

**Deposited data**		

Data	Zenodo	https://zenodo.org/records/10201777
Code	Zenodo	https://zenodo.org/records/10201777

**Experimental models: Organisms/strains**		

CEN.PK113-5D	P. Kotter (Frankfurt, Germany)	N/A
CEN.PK113-5D + pDRF1-GW	W. Frommer	Addgene #36026
CEN.PK113-5D + tdTomato pDRF1-GW	D. Botman	In-house
CEN.PK113-5D + yAT1.03 pDRF1-GW	D. Botman	Addgene #132781
CEN.PK113-5D + yAT1.03R122KR126K pDRF1-GW	D. Botman	Addgene #132782
CEN.PK113-5D + ymTq2 pDRF1-GW	D. Botman	Addgene #118453
CEN.PK113-5D + ymTq2Δ11 pDRF1-GW	This paper	This paper; Addgene #205490
CEN.PK113-7D	Kotter et al.^35^	https://doi.org/10.1016/S0580-9517(06)36025-4

**Oligonucleotides**		

FW 5' - TAAGCAGCTAGCAC TAGTAAGCTTTTAA - 3′	This paper; Thermo Fisher Scientific, Scientific, Waltham, MA, USA	N/A
RV 5' - TAAGCAGCGGCCGCTTA AGCAGCAGTAACGAATTCC - 3′	This paper; Thermo Fisher Scientific, Scientific, Waltham, MA, USA	N/A

**Software and algorithms**		

flowCore	Ellis et al.^36^^,^^37^	https://bioconductor.org/packages/flowCore
ImageJ	Schneider et al.^38^	https://imagej.net/ij/
FiJi	Schindelin et al.^39^	https://imagej.net/software/fiji/
Package ‘arules’	Hahsler et al.^40^	https://cran.r-project.org/web/packages/arules/arules.pdf
R Core Team	R Foundation for Statistical Computing, Vienna, Austria	http://www.R-project.org/
Trainable Weka Segmentation	Arganda-Carreras et al.^41^	https://imagej.net/plugins/tws/
TurboReg	Thevenaz et al.^42^	https://imagej.net/plugins/turboreg

**Other**		

ConA-coated coverslips	Hansen et al.^43^	https://doi.org/10.1038/nprot.2015.079
DNA sequencing	Macrogen Europe BV, Amsterdam, The Netherlands	https://www.macrogen-europe.com/
Dried membrane filters 0.4 μm	Whatman	CAS: 10417106
Dropout mix	Cold Spring Harbor Protocols	https://doi.org/10.1101/pdb.rec8585
PES filter 0.2 μm	Santa Cruz Biotechnology Inc., Dallas, TX, USA	CAS: sc-516079
Transformation protocol	New England Biolabs, Ipswich, Massachusetts, USA	https://international.neb.com/protocols/2012/05/21/transformation-protocol
Verduyn media	van Dijken et al.^44^	https://doi.org/10.1099/00221287-136-3-395
Yeast transformation protocol	Tutucci et al.^45^	https://doi.org/10.1101/2022.03.01.481833

### Resource availability

#### Lead contact

Further information and requests for resources and reagents should be directed to and will be fulfilled by the lead contact, Bas Teusink (b.teusink@vu.nl).

#### Materials availability

The plasmid ymTq2Δ11 generated in this study have been deposited to [Addgene, pDRF1-GW ymTq2-d11, #20549].

#### Data and code availability

Data
  • Data have been deposited at Zenodo and are publicly available since December 10, 2023. Zenodo Data: https://zenodo.org/records/10201777.
Code
  • Code have been deposited at Zenodo and are publicly available since December 10, 2023. Zenodo Code: https://zenodo.org/records/10201777.

### Experimental model and study participant details

The *Saccharomyces cerevisiae* strains CEN.PK113-7D (MATa, URA3, HIS3, LEU2, TRP1, MAL2-8c, SUC2)^35^ and CEN.PK113-5D (MATa, ura3-52, HIS3, LEU2, TRP1, MAL2-8c, SUC2) were used in this work. The yeast strains were selected to harbor different plasmids, according to the purpose of the experiment (Table 1). All cultures were grown in Yeast Nitrogen Base (YNB) media buffered to pH 5 and supplemented with a carbon source of choice (glucose or ethanol), at 200 rpm and 30°C.

*E. coli* DH5-alpha was the bacterial strain selected for the cloning procedures. Cells were grown in Luria-Bertani (LB) broth at 200 rpm and 37°C.

### Method details

#### ymTq2Δ11 construction

The following construction was performed with the purpose of creating the exact same acceptor FP (lacking the last 11 amino acids) incorporated in the yAT1.03 sensor and in the correspondent non-responsive variant. pDRF1-GW ymTq2Δ11 was generated by removing the yAT.03 from pDRF1-GW yAT1.03 and ligating the opened vector with the ymTq2 lacking the last 33 nucleotides. To amplify ymTq2Δ11, the following primers were used: FW- TAAGCAGCTAGCACTAGTAAGCTTTTAA and RV- TAAGCAGCGGCCGCTTAAGCAGCAGTAACGAATTCC, containing restriction sites for *Nhe*I and *Not*I, respectively. The PCR was performed with Phusion Hot Start II DNA Polymerase (2 U/μL) (Thermo Fisher Scientific, Scientific, Waltham, MA, USA) according the manufacturer specifications. A temperature of 55°C was used for the annealing of the primers. The PCR product was first purified using the GeneJET PCR Purification Kit (Thermo Fisher Scientific) to concentrate the DNA. A second purification step was performed in a 1% agarose gel using the GeneJET Gel Extraction Kit (Thermo Fisher Scientific) to remove the original vector used as template in the PCR reaction. pDRF1-GW yAT1.03 and ymTq2Δ11 were digested with *Nhe*I HF and *Not*I HF (New England Biolabs, Ipswich, Massachusetts, USA) and the opened vector pDRF1-GW and insert ymTq2Δ11 purified from a 1% agarose gel using the GeneJET Gel Extraction Kit. The vector and the insert were ligated using T4 DNA Ligase (Thermo Fisher Scientific) and transformed in *E. coli* cells. The transformation was performed in chemocompetent cells according the Transformation Protocol of New England Biolabs (https://international.neb.com/protocols/2012/05/21/transformation-protocol). Positive candidates were selected for plasmid DNA isolation using the GeneJET Plasmid Miniprep Kit (Thermo Fisher Scientific). The construction pDRF1-GW ymTq2Δ11 was confirmed by DNA sequencing (https://www.macrogen-europe.com/). The new plasmid was introduced in CEN.PK113-7D by yeast transformation (performed according to the protocol described by Tutucci E. et al.^45^). Yeast cells were selected in a plate of YNB medium (6.8 g/L Yeast Nitrogen Base (Sigma-Aldrich, St. Louis, MO, USA) and 20 g/L SeaKem LE agarose (Lonza)) -uracil Drop Out^46^ supplemented with 100 mM glucose (Boom BV, Meppel, Netherlands). Fluorescence was confirmed by microscopy.

#### Plate reader growth experiments

A single colony of each strain (7D, 5D, yAT1.03, yAT1.03NR, tdTomato and ymTq2Δ11) (Table 1) was inoculated in 5 mL of YNB media (6.8 g/L Yeast Nitrogen Base and 10.2 g/L potassium hydrogen phthalate (Sigma-Aldrich), adjusted to pH 5 with KOH) supplemented with 100 mM glucose and grown at 30°C and 200 rpm until biomass became visible. Cells were diluted twice in fresh media and kept in mid-log phase until they were collected for the experiment. Cells were then washed twice in YNB supplemented with 10 mM glucose (4000 rpm at room temperature during 2 min), and resuspended in the same media to an ${\text{OD}}_{600}$ of 0.05. A volume of 500 μL of the cell suspension was transferred to a 48 wells plate and the ${\text{OD}}_{600}$ was measured in a FLUOstar Omega microplate reader every 5 min. Cells were kept at 30°C and 700 rpm orbital shaking.

#### Diauxic shift growth conditions and sampling

Cells from a single colony (yAT1.03, yAT1.03NR, tdTomato and ymTq2Δ11) (Table 1) were inoculated in YNB medium supplemented with 100 mM glucose and grown at 30°C and 200 rpm to mid-log phase. Cells were washed twice in YNB medium supplemented with 10 mM glucose (4000 rpm at room temperature) and grown overnight. Next day, growth was monitored by measuring the ${\text{OD}}_{600}$ of the culture, using an Ultraspec 2100 pro (Amersham Biosciences, Cambridge, England). Samples were taken for flow cytometry and metabolites concentrations. The second were filtered with a 0.2 μm polyethersulfone (PES) filter and kept at −20°C until further use.

#### Microscopy growth conditions

Yeast cultures (yAT1.03 and yAT1.03NR) (Table 1) were grown in YNB media supplemented with 1% ethanol (VWR International, Radnor, PA, United States of America) or 100 mM glucose at 200 rpm and 30°C to mid-log phase. Ethanol pre-grown cells where initially grown in YNB supplemented with 1% ethanol and 5 mM glucose to speed up growth. Cells where then transferred to the same media with ethanol as the sole carbon source and later collected for the AA and 2-DG pulse experiments performed in the microscope.

#### Chemostat growth conditions and sampling

Chemostat cultures of CENPK-113-5D expressing yAT1.03 (Table 1) were grown at 30°C in a 1.2 L bioreactor (Applikon, Delft, the Netherlands) with a maintained working volume of 0.5 L and a stirrer speed of 500 rpm. Media was prepared as described by Verduyn et al.^44^ and supplemented with 10 mM glucose as carbon source and additional 0.2 g/L of antifoam C (Sigma-Aldrich). Dissolved oxygen concentration was maintained above 40% throughout the cultivation. Steady-state was assumed after eight generation times. After the initial batch phase, indicated by rise in oxygen levels in the off-gas, pumps were turned on to maintain a dilution rate of 0.1 $h^{-1}$ for the first sampling. The dilution rate was later increased to 0.25 $h^{-1}$ for the second sampling. Chemostat samples were diluted with spent media, filtered with a 0.2 μm PES filter, to an ${\text{OD}}_{600}$ of 1 at room temperature. Samples were taken from the diluted cell suspension for flow cytometry. During the time span of the flow cytometry analysis, sub-samples from the original cell suspension were collected through a 0.2 μm PES filter (Whatman, Santa Cruz Biotechnology Inc., Dallas, TX, US) and stored at −20°C for subsequent glucose concentration measurements. Cell dry weight was determined by filtering 5 mL of the culture on weighed and dried membrane filters of 0.4 μm (Whatman). Filters were washed with demi water and dried at 60°C for 24 h prior to weighing.

#### Metabolites concentration determination

Glucose concentrations from the chemostat samples were determined enzymatically with a solution of hexokinase/glucose-6-phosphate dehydrogenase (Roche) in PIPES buffer at pH 7. Measurements were performed in a FLUOstar Omega microplate reader. After addition of the enzymes, samples were incubated for 15 min and absorbance was measured at 340 nm. Glucose concentrations were determined by comparison of the endpoint measurements with a glucose calibration curve. The calibration curve was measured as described above with known glucose concentrations and in parallel with the samples. All measurements were performed in triplicate and the background absorbance was measured from samples without enzyme addition. Additional metabolites were measured by High-Performance Liquid Chromatography (HPLC). This was also the case for samples from the batch cultures collected during the diauxic shift experiment, including glucose. Such samples were thawed at 4°C O/N and measured next day on a prominence HPLC (SHIMADZU, Kyoto, Japan) equipped with an Rezex ROA organic acid $H^{+}$ column (Phenomex, CA, USA). A post-run analysis (LabSolutions v5.71) using calibration curves with known concentrations and retention times was performed to retrieve the metabolites concentrations. Four (yAT1.03 and yAT1.03NR) or two (tdTomato and ymTq2Δ11) independent cultures were included in the analysis.

#### Flow cytometry

The ATP levels in yAT1.03 cells were followed in a flow cytometer CytoFLEX S (Beckman Coulter, Brea, USA) using the software CytExpert (v2.4.0.28). The flow rate was set to 10 μL/min and constrained to 2000 events/sec. A violet laser was used in combination with the RFP-405nm (610/20) and CFP-405nm (470/20) filters. A tube containing 920 μL of the cell suspension was placed in the machine and the baseline measured for 2 min. A volume of 100 μL of 0.5 mM AA diluted in 5% ethanol was added to the cells and mixed with a pipette. Alternatively, cells were pulsed with 100 μL of 0.5 M 2-DG diluted in demi water. The response was measured for additional 5 min. Samples were processed using the slow speed running mode. In between samples we performed a first 1 min wash with absolute ethanol (to remove possible AA residues in the machine) and a second 2 min wash with demi water (to wash the ethanol) using the fast speed running mode. In order to correct the fluorescence of tdTomato for ymTq2Δ11 bleedthrough, we measured the fluorescence in the RFP-405nm and CFP-405nm channels of the strain ymTq2Δ11 at different time points of the diauxic shift and applied the average. The yeast strain yAT1.03NR was used as a negative control. A minimum number of 10.000 events was considered.

#### Microscopy

Cells harboring yAT1.03 or yAT1.03NR sensors were transferred to a Attofluor cell chamber (Thermofisher Scientific, Waltham, MA, USA), containing a ConA-coated coverslip (prepared in PBS according Hansen et al.^43^), and incubated at 30°C for 15 min, following two washing steps in 900 μL of fresh media. Ethanol pre-grown cells were washed in YNB media 1% ethanol and glucose pre-grown cells in YNB 10 mM glucose. Cells were placed in a Nikon Ti-eclipse widefield fluorescence microscope (Nikon, Minato, Tokyo, Japan) at 30°C for imaging. A 2 min baseline was recorded before the addition of 100 μL of Antimycin A (Sigma-Aldrich; final concentration 50 μM in ethanol 0.5%) or 2-DG (final concentration 10–100 mM). After the pulse, the response was followed for additional 8 min. Cells were imaged every 20 s and FRET was recorded using a TuCam system (Andor, Belfast, Northern Ireland) equipped with 2 Andor Zyla 5.5 sCMOS cameras (Andor). A 438/24 nm excitation filter, a 483/32 nm donor emission filter and a 593/40 nm acceptor emission filter (552 nm long-pass dichroic filter) (Semrock, Lake Forest, IL, USA) were used. The light intensity was adjusted to 7.4 in a SOLA 6-LCR-SB power source (Lumencor, Beaverton, OR, USA). Cell segmentation was performed using an in-house macro in FiJi (NIH, Bethesda, MD, USA).^39^ First, image drift across channels was corrected in ImageJ^38^ using the 2D image stabilizer plugin TurboReg^42^ developed by P. Thevenaz et al. and background correction was performed. Next, cells were segmented using FiJi Trainable Weka Segmentation plugin^41^ developed by Arganda-Carreras et al. and the brightest channel (CFP) as reference. The mean fluorescence of each cell was calculated for both fluorescent channels. A filter based on size and round shape was applied to remove cell aggregates (min size of 2000 or 3000 pixels depending on the dataset and max size of 1200 pixels; 0.6–1 roundness).

### Quantification and statistical analysis

R Core Team (2013) was used for data analysis and visualization. R: A language and environment for statistical computing. R Foundation for Statistical Computing, Vienna, Austria. ISBN 3-900051-07-0, URL http://www.R-project.org/. The R package flowCore^36^^,^^37^ was used for cell gating and filtering. The fluorescence of tdTomato was corrected for ymTq2Δ11 bleedthrough (0.774±0.091 of ymTq2Δ11 fluorescence) and the normalized FRET ratio was calculated as RFP/CFP (or tdTomato/ymT2Δ11) regularized to the baseline (before the addition of the compound, and considered until 1.3 and 2 min for the flow cytometry and microscopy analyses, respectively). The distribution plots of the ATP dynamics after AA addition include the data of the second half of the experiment (3.33 min from the start). The FRET ratio illustrated in the binned plot was calculated using the discretize function of the R package arules^40^ and 50 breaks. To calculate the ATP consumption rate we applied a breakpoint analysis using segmented regressions.^47^ To determine the biomass specific rates, a rolling window with 6 constitutive measurements was used. The biomass specific rate was calculated by multiplying the growth rate by the metabolite yield on biomass. The biomass and metabolites concentrations experimental data were fitted by splitting it into two subsets, before and after glucose exhaustion, and fitting each of the new subsets independently. The substrate yield on biomass was calculated by linear regression on the biomass concentration (OD600) versus metabolite concentration (mM). The growth rate was calculated by applying linear regressions on the natural logarithm of time (h) versus biomass concentration (OD600). Statistical analysis was performed using R, and results are presented as violin plots or mean ± SD. Comparisons among replicates were calculated using the statistical tests Mann-Whitney U (p value <0.0001) and Analysis of Variance ANOVA. In most plots we chose to plot the replicates data independently or instead, to include a representative replicate in the main manuscript and the additional replicates in the supplements.
