# Supplementary material for: A fast method to distinguish between fermentative and respiratory metabolisms in single yeast cells
Source: iScience. 2023 Dec 21;27(1):108767. doi: 10.1016/j.isci.2023.108767 (PMC10793178; doi:10.1016/j.isci.2023.108767)
Supplement: Document S1. Figures S1–S11 and Tables S1 and S2 [file mmc1.pdf]

iScience, Volume *27*

Supplemental information

A fast method to distinguish between fermentative and respiratory metabolisms in single yeast cells

Laura Luzia, Julius Battjes, Emile Zwering, Derek Jansen, Chrats Melkonian, and Bas Teusink

The fluorescent protein ymTq2 was modified by removing its last 11 amino acids (ymTq2Δ11) in order to accurately mimic the acceptor protein present in the FRET sensor yAT1.03 used in this work, and previously described by Botman *et al.*[1]. The fluorescence of the new ymTq2Δ11 cyan variant was tested by microscopy (Figure S1 a). To correct the flow cytometry data for the bleedthrough of the acceptor FP (ymTq2Δ11) into the donor FP (tdTomato), we measured the fluorescence of the strain CEN.PK113-5D + ymTq2Δ11 in the blue and red channels during the diauxic shift (Figure S1 b). No differences were observed in the fluorescence ratio Red/Blue over the course of the diauxic shift experiment (Figure S1 c). The mean value was used for correction purposes of the tdTomato fluorescence.

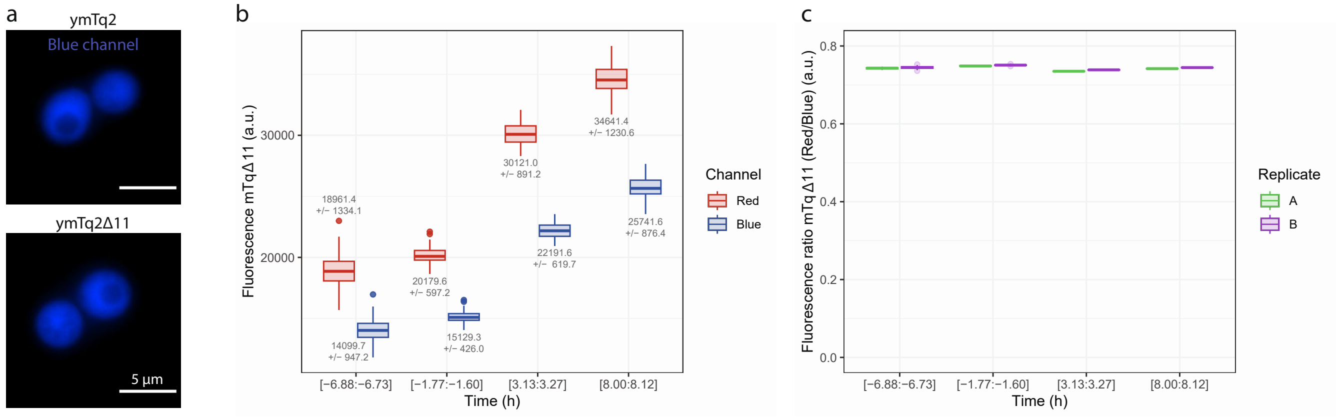

Figure S1. Fluorescence emitted by CEN.PK113-5D + ymTq2Δ11 in the red and blue channels and respective ratio during a diauxic shift experiment, related to Figure 1. (a) Yeast cells expressing ymTq2 or ymTq2Δ11 were pre-grown on glucose and visualized using widefield microscopy. (b) Fluorescence of cells expressing ymTq2Δ11 was measured in the red and blue channels by flow cytometry and the respective ratio calculated. Data combines replicates A and B. (c). Despite the increase in fluorescence observed in the two channels, the contribution was the same in both, resulting in a constant ratio over time. Data plotted in c) was discretized to 50 breaks and shows the replicates separately. Time 0 h corresponds to the diauxic shift.

The growth profiles of the yeast strains described in the present work (Table 2) were followed over time (Figure S2). We observed similar growth rates on glucose across strains, however slight differences were found for cells growing on ethanol, with the prototrophic strain CEN.PK113-7D and the strain expressing the empty vector pDRF1 displaying the highest growth rate and maximum final biomass reached.

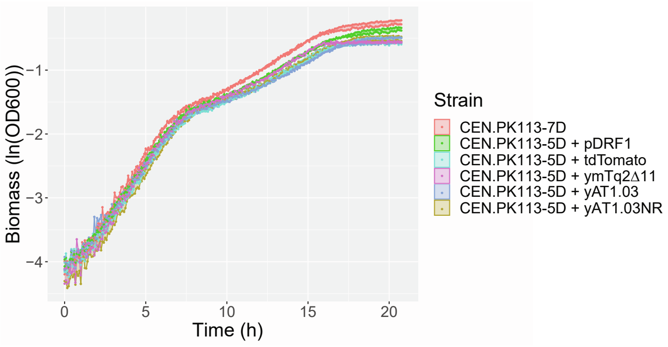

Figure S2. Growth profiles of the yeast strains used in this work, related to Figure 1. Cells were grown in YNB media supplemented with 10 mM glucose. The prototrophic strain CEN.PK113-7D and the strain CEN.PK113-5D + pDRF1 were used as controls. Data include two biological replicates and two technical replicates per experiment.

We started by measuring the *in vivo* response of the sensor yAT1.03 to 10 mM of 2-Deoxy-D-glucose (2-D) (same equimolar concentration to the glucose available), as described by Botman *et al.*[1]. However, we found that this concentration was insufficient to fully block ATP synthesis through glycolysis in CEN.PK113-5D (Figure S3). As a result, we selected a concentration of 50 mM 2-DG for this study.

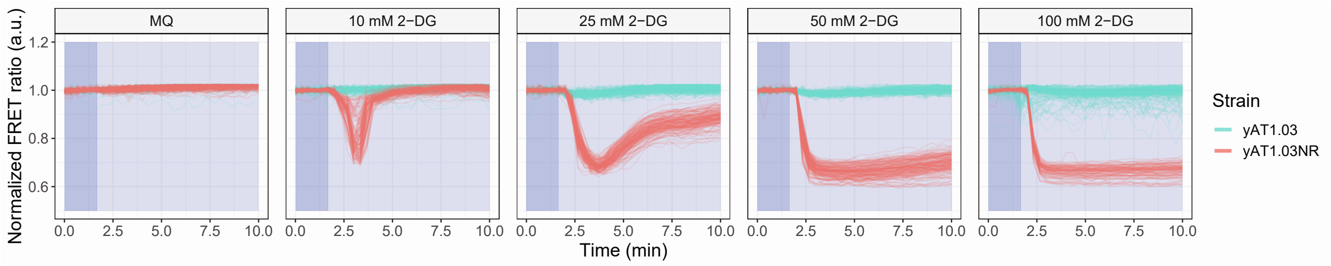

Figure S3. ***In vivo* dose response of yAT1.03 to 2-Deoxy-D-glucose, related to Figure 1.** Cells pre-grown on 100 mM glucose were washed and incubated in 10 mM glucose followed by addition of 2-DG (min 2). Fluorescence was measured by widefield microscopy, allowing to track individual responses, here represented by a thin line. The FRET ratio was normalized to the baseline (dark blue area) and plotted over time.

ATP dynamics of two independent chemostat cultures, harboring yAT1.03 and grown in fermentative or respiratory regimes (D=0.25 and 0.1 h^{-1}, respectively) after the addition of 2-Deoxy-D-glucose (Figure S4). Cells were harvested from the chemostat in a 50 mL tube, and an aliquot was transferred to a microcentrifuge tube for flow cytometry. An unexpected drop in signal in replicate A was observed at 0.1 h^{-1} before the addition of the compound, for which we currently lack a plausible explanation. Despite this initial drop, the FRET ratio was constant 1 minute before the compound addition.

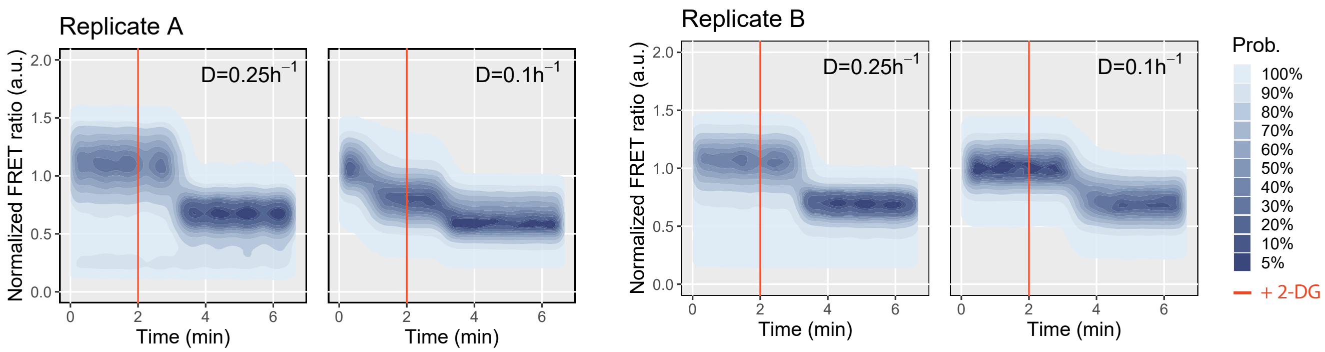

Figure S4. Response of cells grown in a chemostat to a 2-Deoxy-D-glucose pulse, related to Figure 1. Cells expressing yAT1.03 were grown in a chemostat at 0.1 and 0.25 h^{-1} followed by addition of 50 mM 2-DG (min 2). FRET ratio measured by flow cytometry and normalized to the baseline. Data is originated from two biological replicates (A and B) and replicate B was selected to be displayed in the main manuscript.

We monitored the glucose concentration over time of the sampled chemostat broth used in the pulse experiment with yAT1.03 cells performed in the flow cytometer (2-DG and AA) (Figure S5 and Table S2). Data show that in both cases there was glucose present during the pulse experiment. Glucose concentrations ranged between 10 and 5.5 µM and between 67 and 50 µM for the 0.1 and 0.25 h^{-1} dilution rates cultures, respectively.

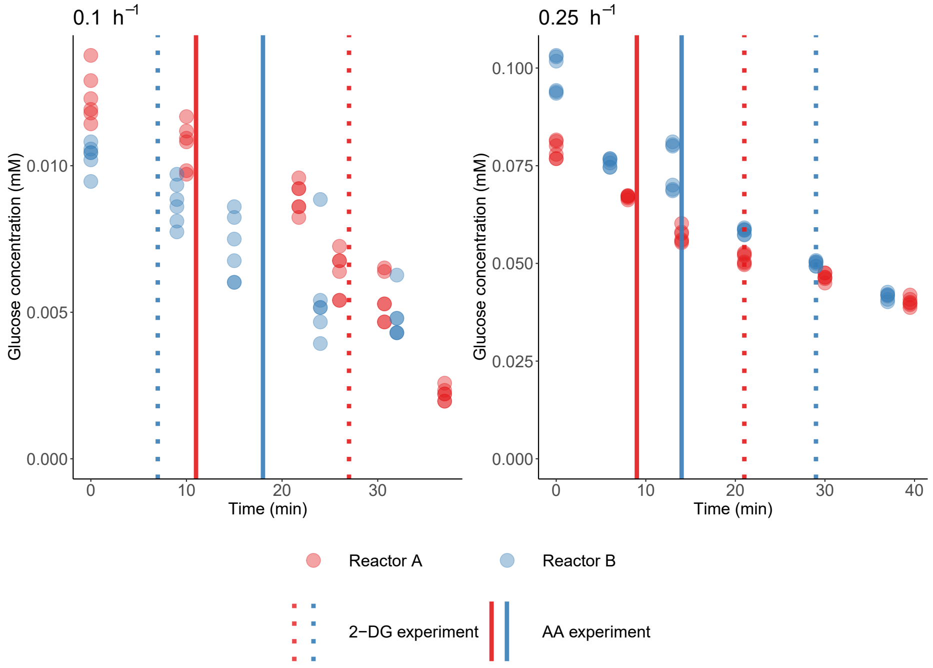

Figure S5. Glucose depletion in chemostat samples, related to Figure 1 and 2. The glucose concentration of the chemostat samples taken for the pulse experiment was monitored through time to confirm that a residual amount would still be present at the moment of the experiment. The dots indicate the measured glucose concentration at the corresponding time point. The lines indicate the time at which either the 2-DG (dashed) or the AA (solid) was added in the flow cytometer experiment for either reactor A (red) or reactor B (blue).

We confirmed the response of yeast cells to Antimycin A addition previously obtained by microscopy by monitoring the ATP dynamics through flow cytometry under the same growth conditions (Figure S6).

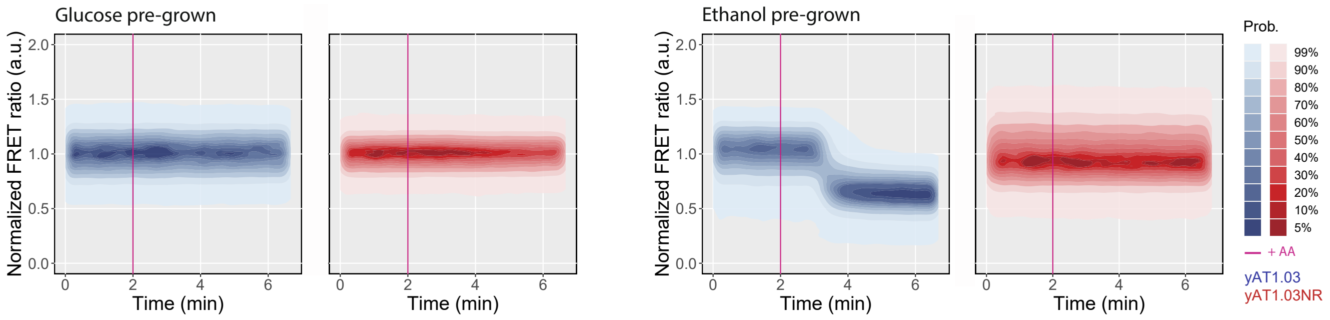

Figure S6. ATP dynamics upon Antimycin A addition to respiratory and fermentative cells, related to Figure 2. Cells expressing yAT1.03 or yAT1.03NR were incubated in YNB media supplemented with 10 mM glucose or 1% ethanol and pulsed with 50 μM of AA. Fluorescence was measured by flow cytometry.

ATP dynamics of two independent chemostat cultures, harboring yAT1.03 and grown in fermentative or respiratory regimes (D=0.25 and 0.1 h^{-1}, respectively) after the addition of AA (Figure S7). Cells were harvested from the chemostat in a 50 mL tube, and an aliquot was transferred to an microcentrifuge tube for flow cytometry. An unexpected drop in signal in replicate A was observed at 0.1 h^{-1} before the addition of the compound, for which we currently lack a plausible explanation. Despite this initial drop, the FRET ratio was constant 1 minute before the compound addition.

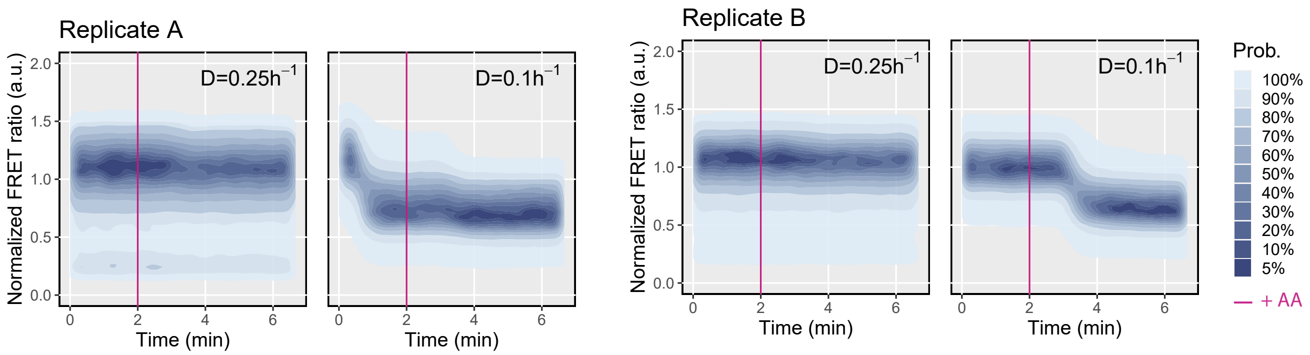

Figure S7. Response of cells grown in a chemostat to an Antimycin A pulse, related to Figure 2. Cells expressing yAT1.03 were grown in a chemostat at 0.1 and 0.25 h^{-1} followed by addition of 50 μM of AA (min 2). FRET ratio measured by flow cytometry and normalized to the baseline. Data is originated from two biological replicates (A and B) and replicate B was selected to be displayed in the main manuscript.

Summary data of cells response to Antimycin A in the different stages of batch growth (glucose, diauxic shift and ethanol) (Figure S8).

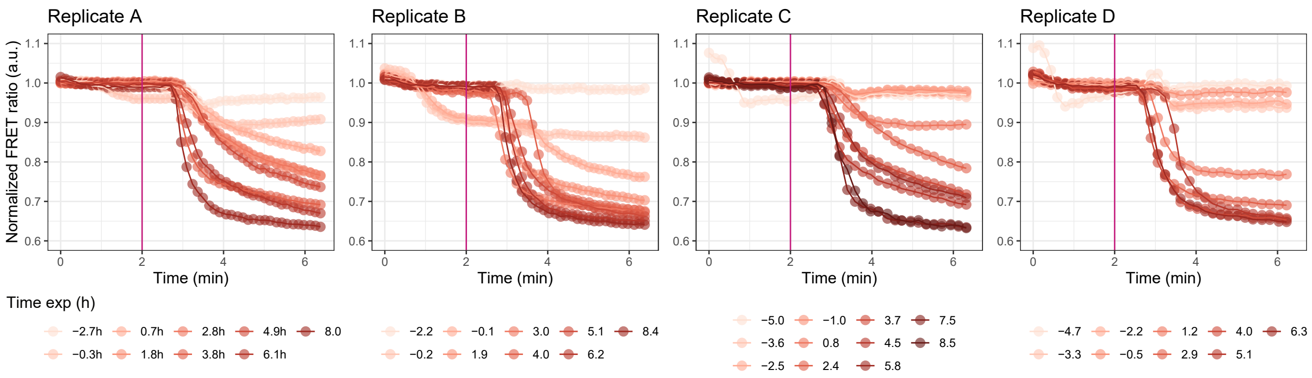

Figure S8. ATP dynamics upon Antimycin A addition to yeast cells pre- during and post-diauxic shift, related to Figure 3. Binned plots of the normalized ATP FRET ratio (mean data of 50 consecutive data points) of yAT1.03 and yAT1.03NR cells after addition of 50 μM of AA. Plots include data of four biological replicates (A, B, C and D). Replicate A was included in the main manuscript.

Throughout the batch experiments we consistently observed an increase in the abundance of a subpopulation with a low normalized FRET ratio (Figure S9, replicate A Figure 3 d). In the absence of carbon source, this subpopulation represents the majority of the cells.

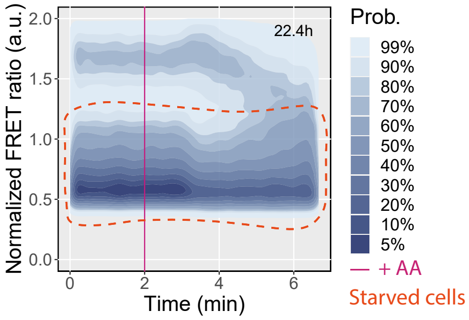

Figure S9. ATP dynamics in yAT1.03 cells post-diauxic shift upon Antimycin A addition reveal a low-ATP subpopulation, related to Figure 3. Starved cells display a low normalized FRET ratio when compared with cells grown on glucose or ethanol and are unable to respond to an AA pulse. The time on the top right corner represents the sampling time relative to the diauxic shift.

When looking at the expression of both fluorescent reporters we observed large differences among yAT1.03 cells during the diauxic shift experiment (Figure S10). The reason for this is related with the nature of the plasmid encoding for the sensor, which is copy-number sensitive. The higher fluorescence during the ethanol phase also suggests a higher sensor expression, which is probably related with a decrease in growth rate and therefore protein accumulation. Additionally, we found differences in the non-normalized FRET ratios during the course of the experiment. Those could be linked to variations in the cytosolic composition that differently affect the donor and acceptor proteins. In all cases the observed differences are corrected by plotting the normalized FRET ratio.

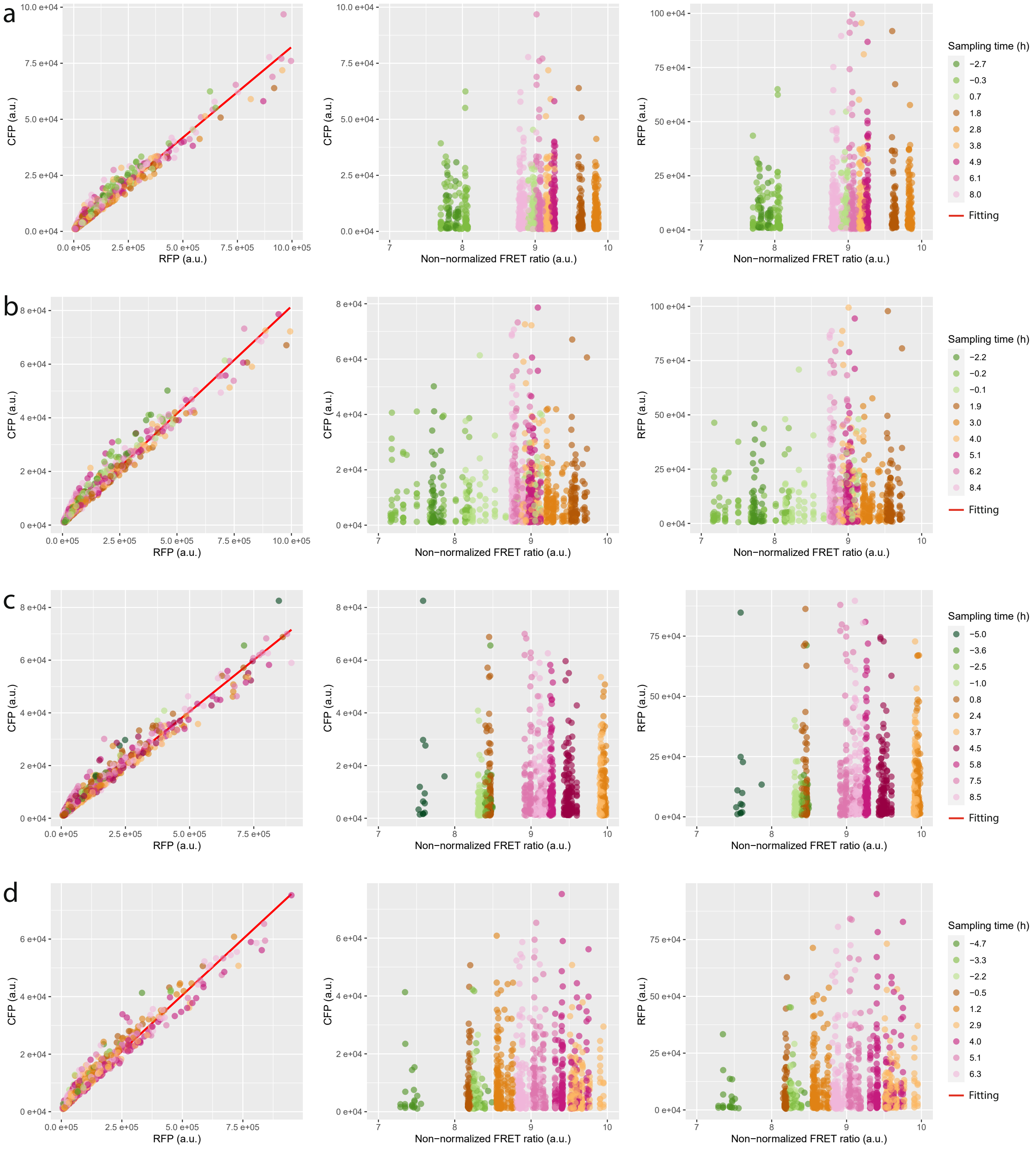

Figure S10. Expression pattern of the fluorescent reports and relationship with the non-normalized FRET ratio, related to Figure 3. Data included corresponds to the diauxic shift experiment represented in Figure S8. Subplots a, b, c and d refer to the four biological replicates (A, B, C and D, respectively). Data was filtered to the baseline (< 2 min) and sampled to 1000 events for a cleaner visualization.

We hypothesised that a higher speed in ATP consumption in response to Antimycin A would positively correlates with the changes in FRET ratio. To assess this, we measured the rate of ATP synthesis inhibition across the diauxic shift experiment and compared it with the difference in normalized FRET ratio before and after the addition of the compound in cells expressing yAT1.03. However we found a strong suggestion that a faster decrease in normalized FRET ratio (indicated by a higher absolute slope) leads to a lower normalized FRET ratio, this was not always the case (Figure S11).

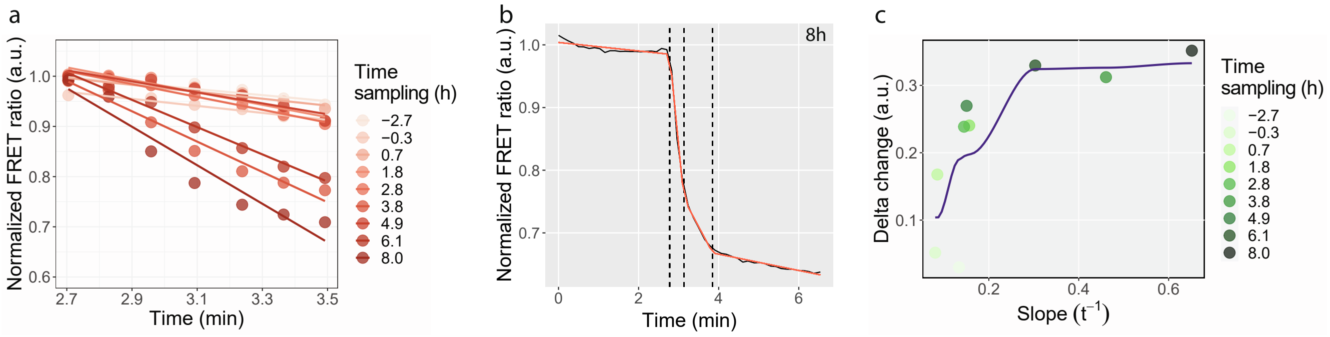

Figure S11. Relationship between maximum ATP synthesis inhibition and delta FRET ratio in Antimycin A pulsed yAT1.03 cells, related to Figure 3. (a) Zoom in on the normalized FRET ratio throughout the diauxic shift experiment (Figure 3 f) between 2.7 and 3.5 min, and respective slopes generated by linear regression. (b) Slopes generated by a breakpoint analysis using segmented regressions for the dataset shown in a) and time experiment 8 h. (c) Delta change of the normalized FRET ratio vs maximum absolute slope. Slopes were calculated according the method displayed in b).

Different methods are available to measure fermentation and respiratory capacity in budding yeast (Table S1). The method here developed, iCRAFT, offers for the first time a quick method to measure single-cell responses to ATP dynamics, allowing to distinguish between the two metabolic regimes.

Among current methodologies, fluorescent dyes stand out as the predominant single-cell approach for assessing membrane potential. Despite their robust discriminatory power between metabolic modes, the method comes with a few disadvantages. Firstly, commonly used cationic dyes require incubation time and are unable to respond to quick changes in membrane potential. Moreover, certain dyes tend to form aggregates, blocking their uniform spread across the mitochondrial matrix, and potentially providing unreliable outcomes[2]. Lastly, fluorescent dyes become diluted with each generation and are challenging to combine with setups where cells and media are continuously replaced, such as chemostats and microfluidics.

Table S1. Current methods to assess yeast respiration, related to Introduction. (i) Refers to intact cells or isolated mitochondria. (ii) Limited to single-cell RNA-sequencing. (iii) Limited in sample size. (iv) The speed of the quantitative analysis will depend on the mitochondrial segmentation pipeline. (v) For statistical purposes, as the assay itself doesn't depend on the cell number.

Method / Reference	Principal	Resolution	Single-cell	Method	Destructive	Type	Time assay	Number of cells
Oxygraph<br>Simonovik *et al.*[3]	Respiration blockage	Quantitative	No	*In vivo*/ *In vitro*	Yes/No (i)	Direct	Few min	4E7
Fluorescent dyes<br>Côrte-Real & Pichová *et al.*[4, 5]	Mitochondria membrane potential	Semi-quantitative	Yes	*In vivo*	No	Direct	15-30 min	1E3 (v)
Omics<br>Cerulus *et al.*[6] & Jariani *et al.*[7]	Quantification of molecules (eg. mRNA)	Quantitative	Yes (ii)/No	*In vitro*	Yes	Indirect	Weeks	2E6
Fluorescent proteins<br>Bagamery *et al.*[8]	Mitochondria morphology (iii)	(Semi-)Quantitative	Yes	*In vivo*	No	Direct	Immediate/Few hours (iv)	1E3 (v)
iCRAFT<br>This work	Respiration blockage	Quantitative	Yes	*In vivo*	No	Direct	Few min	1E3 (v)

Table S2. Depletion of glucose in chemostat samples, related to Figure 1 and 2. The glucose concentrations in the chemostat samples used in the yAT1.03 pulse experiments was followed through time. The mean glucose concentrations per reactor, dilution rates and time points are here represented.

Dilution rate (h^{-1})	Reactor	Time (min)	[Glucose] (µM)
0.1	A	0	12.4
0.1	A	10	10.7
0.1	A	21.75	8.9
0.1	A	26	6.3
0.1	A	30.7	5.5
0.1	A	37	2.2
0.1	B	0	10.3
0.1	B	9	8.7
0.1	B	15	7.2
0.1	B	24	5.5
0.1	B	32	4.8
0.25	A	0	79.1
0.25	A	8	67.0
0.25	A	14	57.1
0.25	A	21	51.2
0.25	A	30	46.5
0.25	A	39.5	40.1
0.25	B	0	98.3
0.25	B	6	75.8
0.25	B	13	74.8
0.25	B	21	58.3
0.25	B	29	50.0
0.25	B	37	41.5

References

1. Botman, D., van Heerden, J. H. & Teusink, B. An improved atp fret sensor for yeast shows heterogeneity during nutrient transitions. *ACS Sensors* **5**, 814–822, DOI: 10.1021/acssensors.9b02475 (2020).
2. Zorova, L. D. *et al.* Mitochondrial membrane potential. *Analytical Biochemistry* **552**, 50–59, DOI: 10.1016/j.ab.2017.07.009 (2018).
3. Simonovik, B. & Gnaiger, E. A mitochondrial reference assay for O2k high-resolution respirometry using freeze-dried baker's yeast. *Mitochondrial Physiology Network* **15** (2017).
4. Ludovico, P., Côrte-Real, M. & Sansonetty, F. Assessment of mitochondrial membrane potential in yeast cell populations by flow cytometry. *Microbiology* **147**, 3335–3343, DOI: 10.1099/00221287-147-12-3335 (2001).
5. Volejníková, A., Hlousková, J., Sigler, K. & Pichová, A. Vital mitochondrial functions show profound changes during yeast culture ageing. *FEMS Yeast Research* **13**, 7–15, DOI: 10.1111/1567-1364.12001 (2013).
6. Cerulus, B. *et al.* Transition between fermentation and respiration determines history-dependent behavior in fluctuating carbon sources. *eLife* **7**, DOI: 10.7554/elife.39234 (2018).
7. Jariani, A. *et al.* A new protocol for single-cell RNA-seq reveals stochastic gene expression during lag phase in budding yeast. *eLife* **9**, DOI: 10.7554/elife.55320 (2020).
8. Bagamery, L. E., Justman, Q. A., Garner, E. C. & Murray, A. W. A putative bet-hedging strategy buffers budding yeast against environmental instability. *Current Biology* **30**, 4563–4578.e4, DOI: 10.1016/j.cub.2020.08.092 (2020).
